# Supplementary material for: Janus-Type AIE Fluorophores: Synthesis and Properties of π-Extended Coumarin-Bearing Triskelions
Source: Molecules. 2022 Nov 2;27(21):7450. doi: 10.3390/molecules27217450 (PMC9656437; doi:10.3390/molecules27217450)
Supplement: Supplementary file 1 [file molecules-27-07450-s001.zip › molecules-1949550-supplementary.pdf]

Supporting Information for

Article

Janus-Type AIE Fluorophores: Synthesis and Properties of π-Extended Coumarin-Bearing Triskelions

Masafumi Ueda [1,*], Mirai Kokubun [1], Nao Yanagi [2], Norifumi Yamamoto [2] and Yasuhiro Mazaki [1,*]

[1] Department of Chemistry, Graduate School of Science, Kitasato University, Kanagawa 252-0373, Japan
[2] Department of Applied Chemistry, Faculty of Engineering, Chiba Institute of Technology, Chiba 275-0016, Japan
* Correspondence: msfmueda@kitasato-u.ac.jp (M.U.); mazaki@kitasato-u.ac.jp (Y.M.)

Contents

1. **^{1}H NMR and ^{13}C NMR Charts** 1
2. **Theoretical calculations** 11
3. **Fluorescence spectra** 13
4. **Absorption spectra** 15
5. **DLS measurement** 16

1. ^{1}H NMR and ^{13}C NMR charts

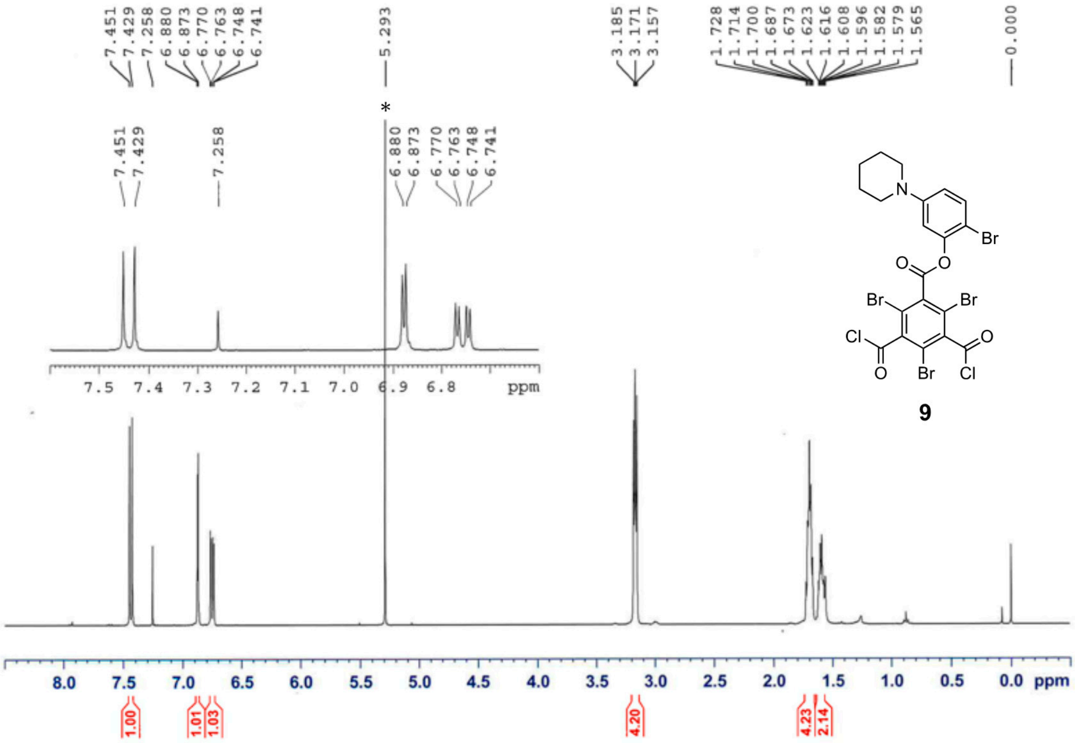

Figure S1. ^{1}H NMR spectrum of **9** (400 MHz, $CDCl_3$, * = solvent peak).

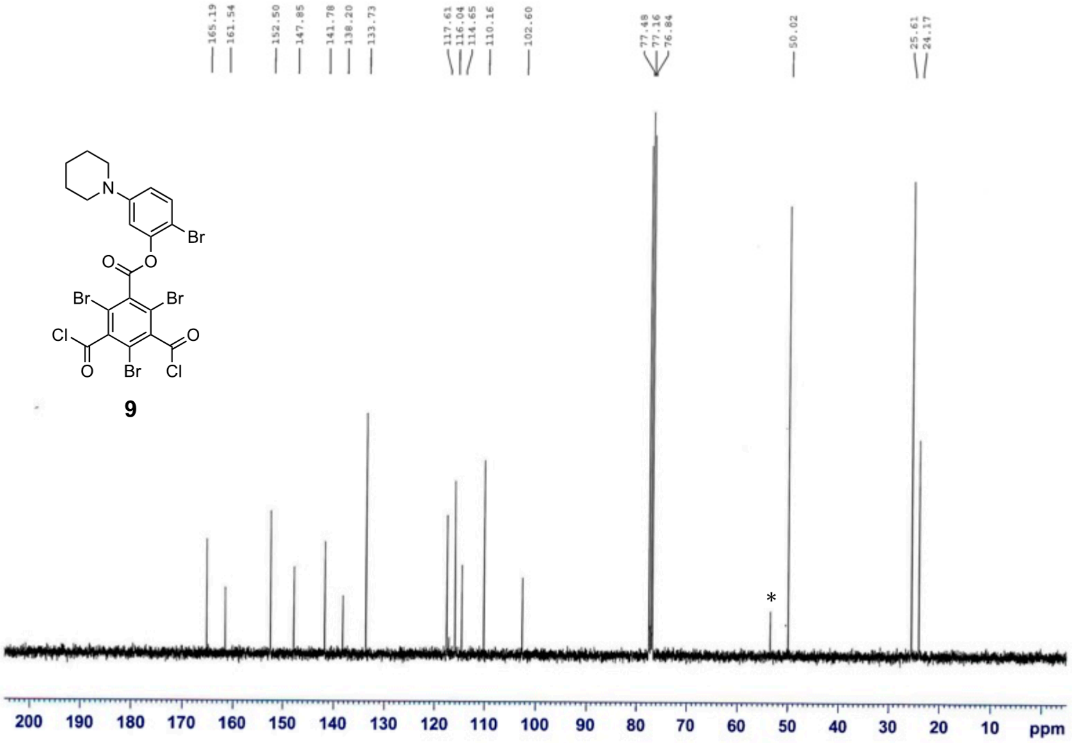

Figure S2. ^{13}C NMR spectrum of **9** (100 MHz, $CDCl_3$, * = solvent peak).

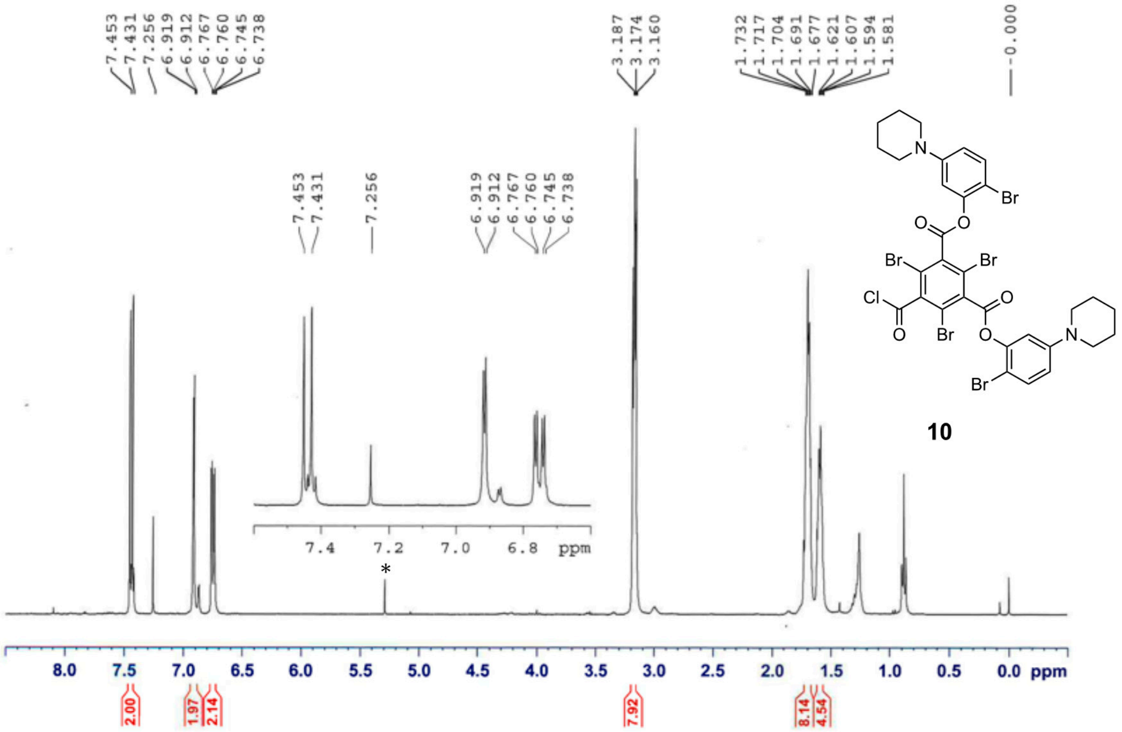

Figure S3. ^{1}H NMR spectrum of **10** (400 MHz, $CDCl_3$, * = solvent peak).

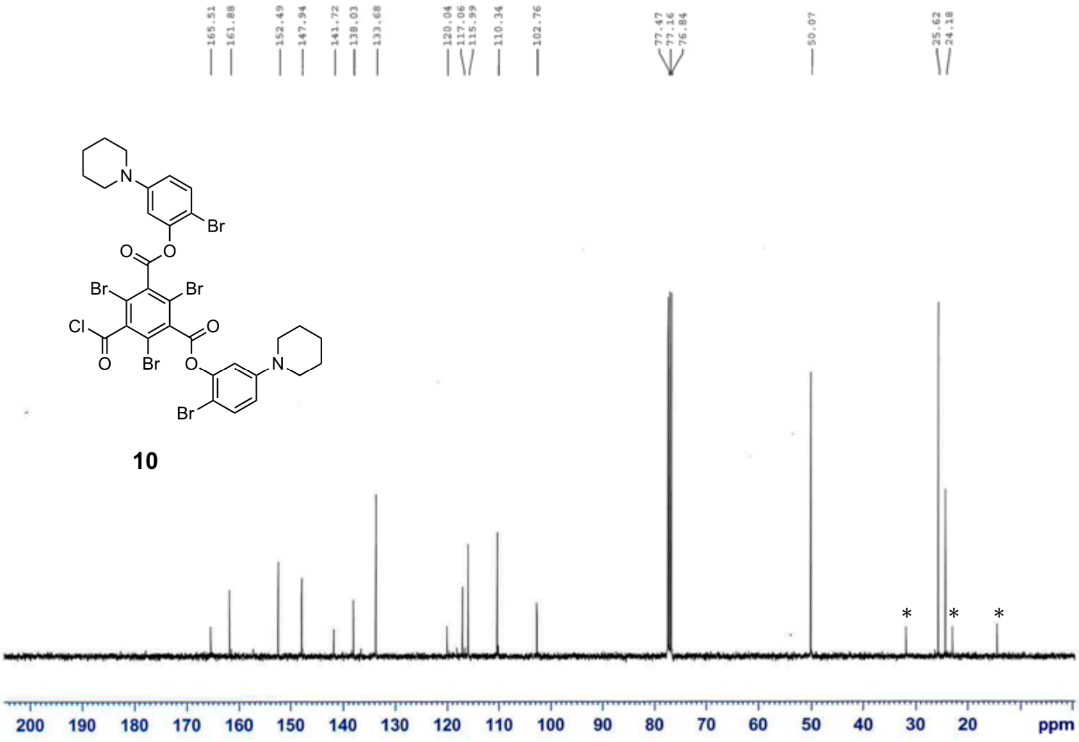

Figure S4. ^{13}C NMR spectrum of **10** (100 MHz, $CDCl_3$, * = solvent peak).

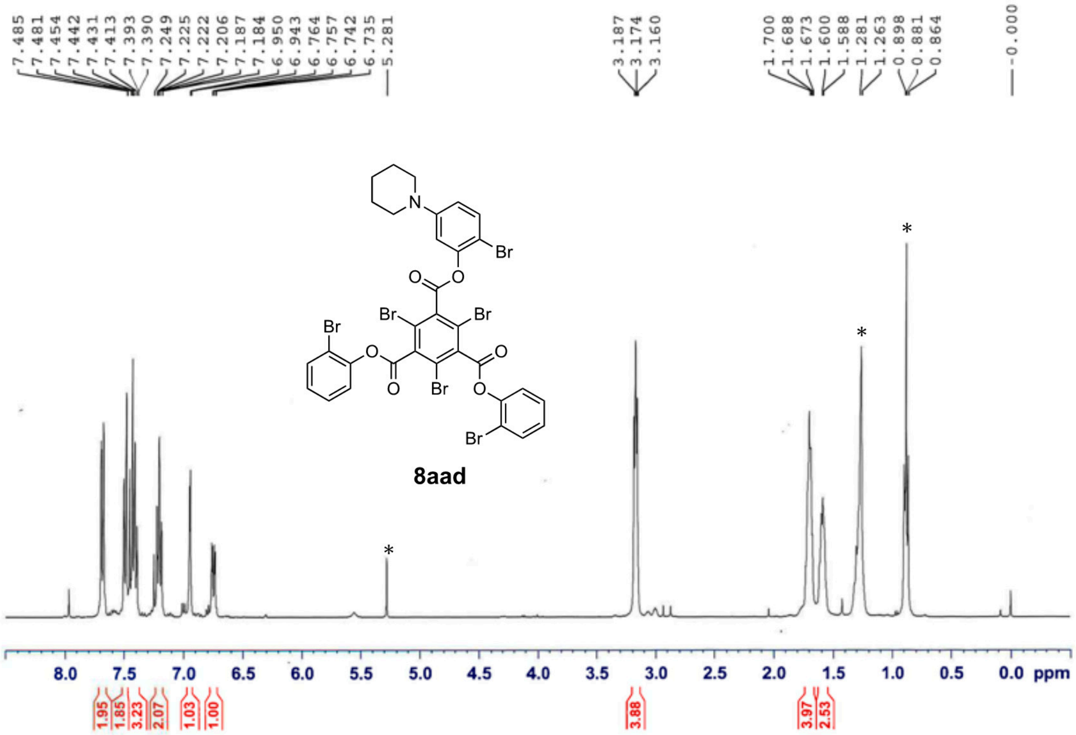

Figure S5. ^{1}H NMR spectrum of **8aad** (400 MHz, $CDCl_3$, * = solvent peak).

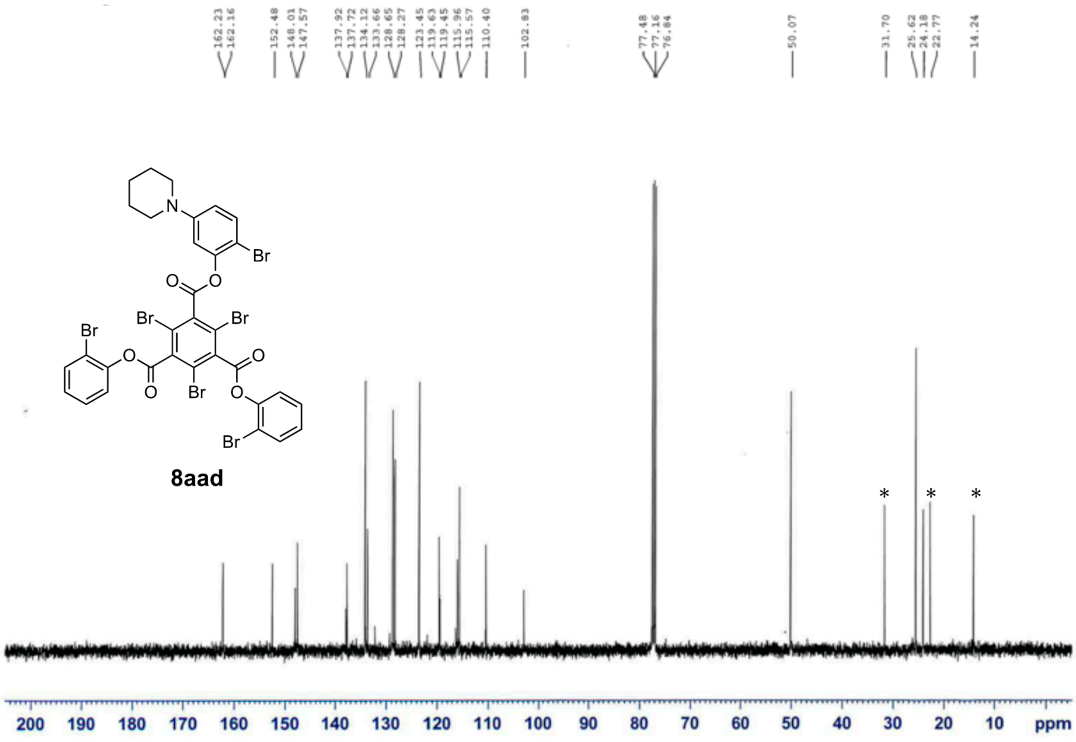

Figure S6. ^{13}C NMR spectrum of **8aad** (100 MHz, $CDCl_3$, * = solvent peak).

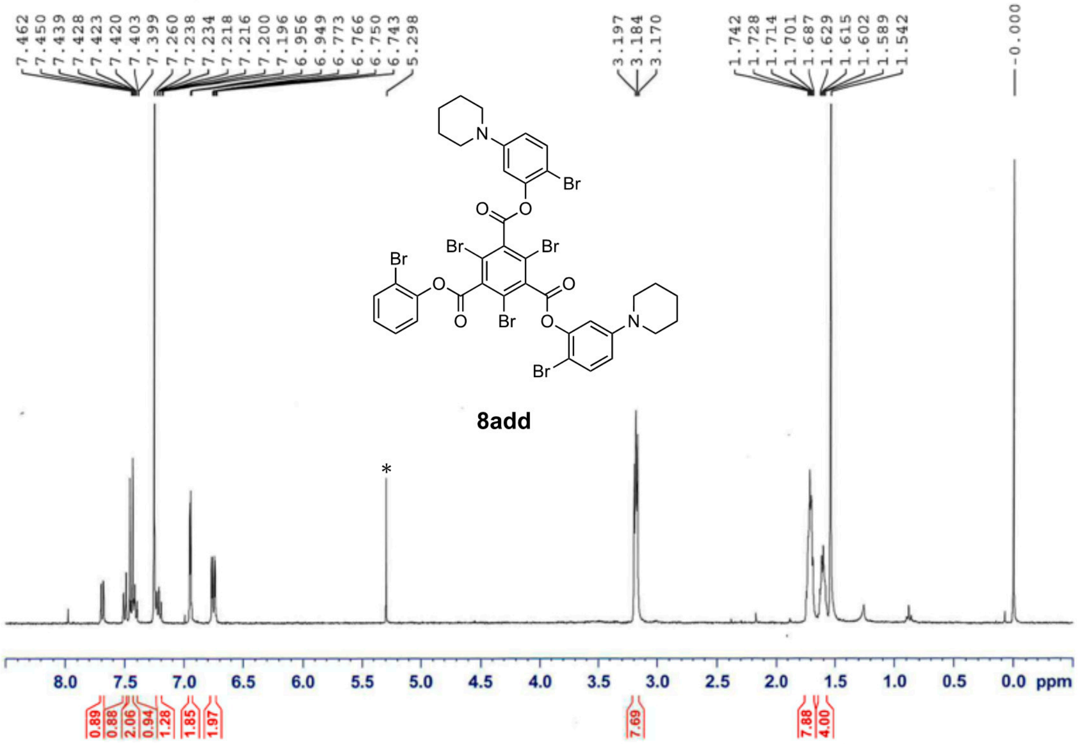

Figure S7. ^{1}H NMR spectrum of **8add** (400 MHz, $CDCl_3$, * = solvent peak).

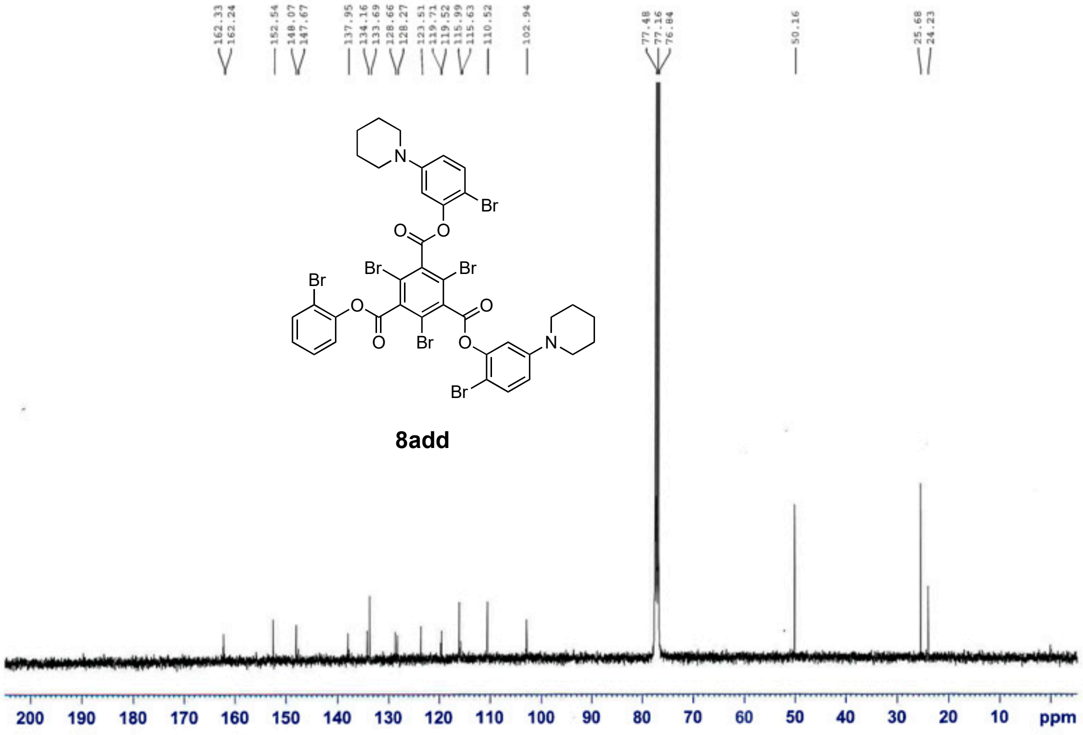

Figure S8. ^{13}C NMR spectrum of **8add** (100 MHz, $CDCl_3$).

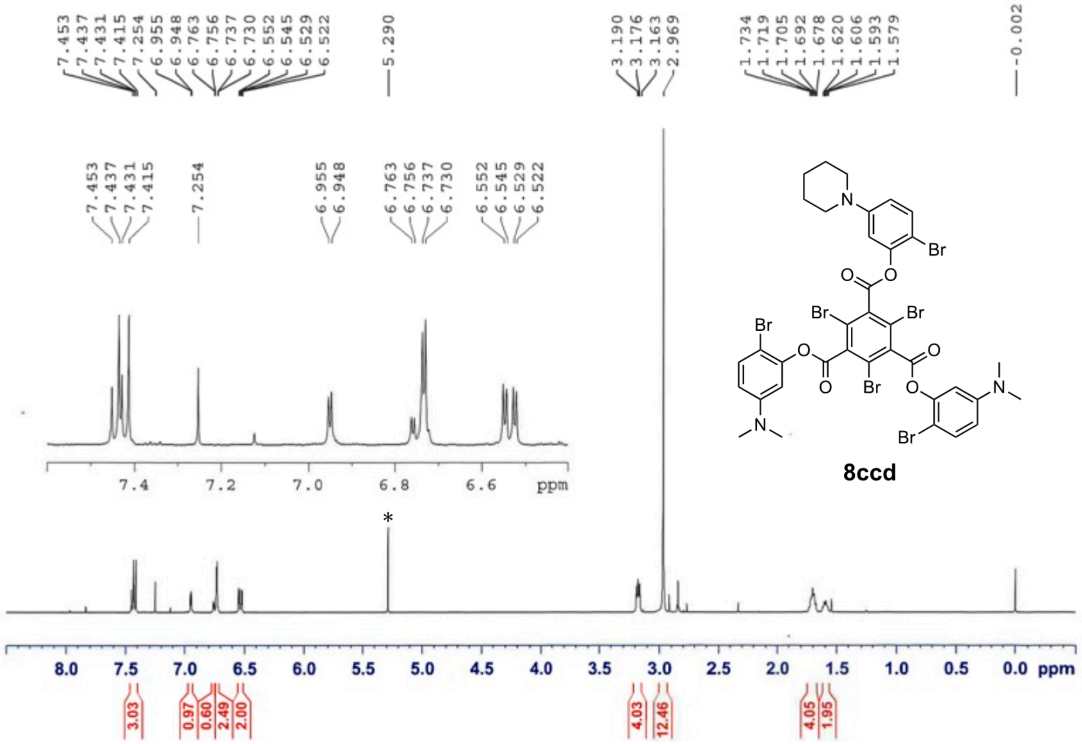

Figure S9. ^{1}H NMR spectrum of **8ccd** (400 MHz, $CDCl_3$, * = solvent peak).

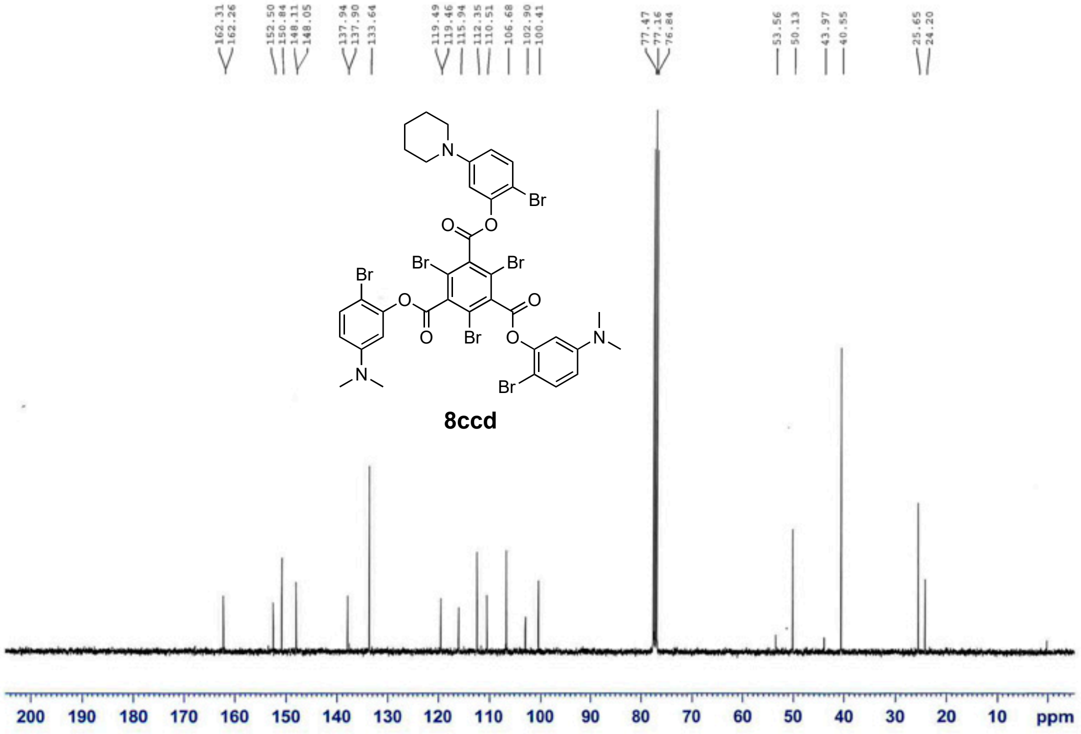

Figure S10. ^{13}C NMR spectrum of **8ccd** (100 MHz, $CDCl_3$).

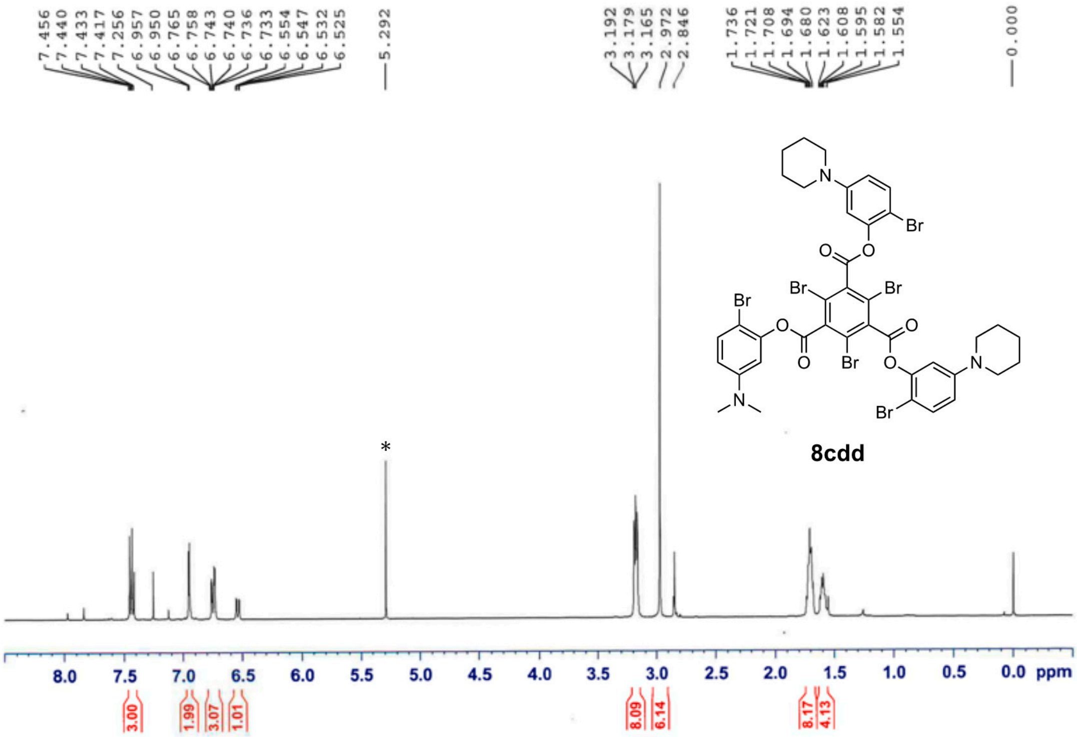

Figure S11. ^{1}H NMR spectrum of **8cdd** (400 MHz, $CDCl_3$, * = solvent peak).

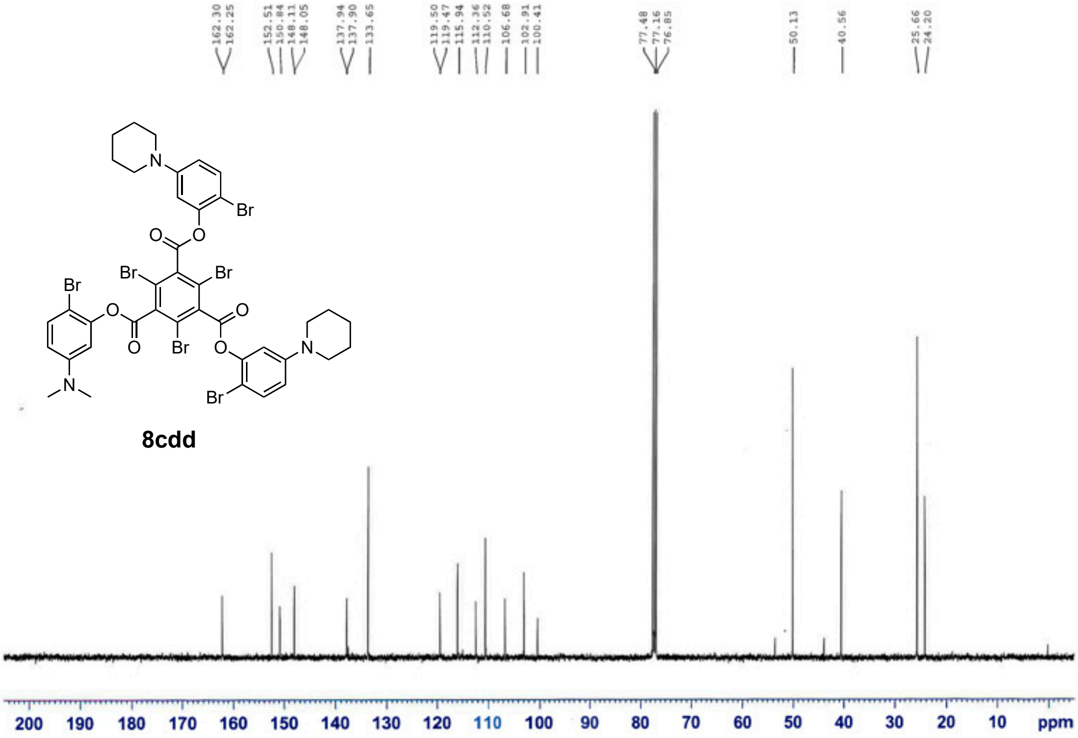

Figure S12. ^{13}C NMR spectrum of **8cdd** (100 MHz, $CDCl_3$).

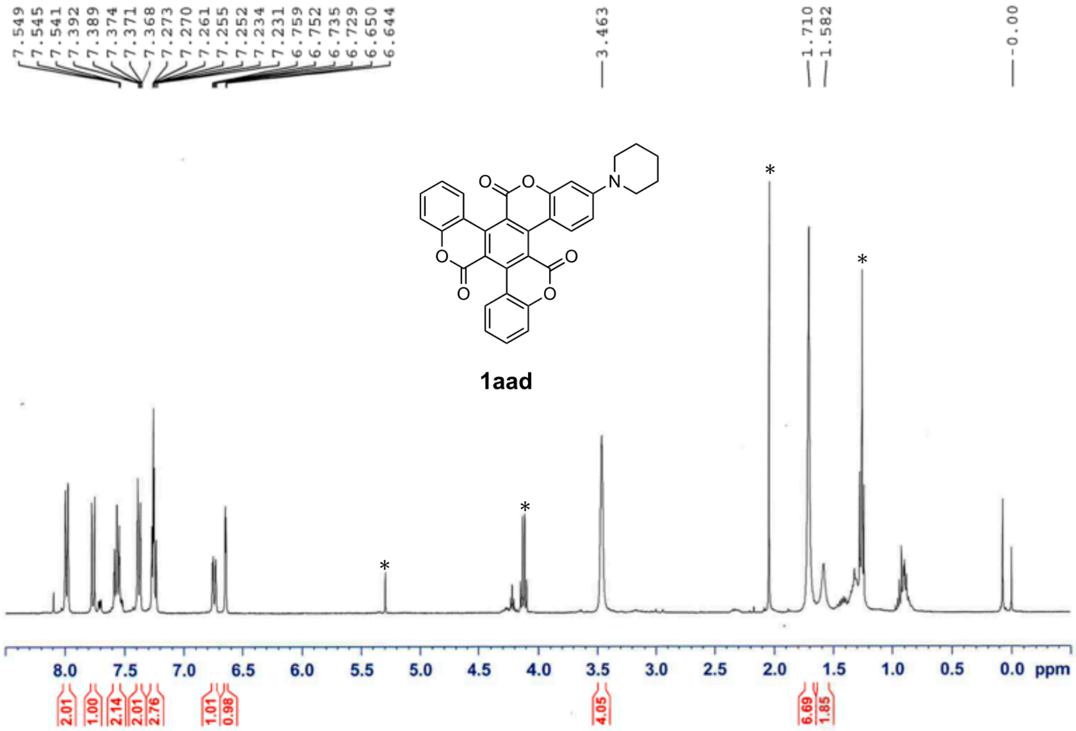

Figure S13. ^{1}H NMR spectrum of **1aad** (400 MHz, $CDCl_3$, * = solvent peak).

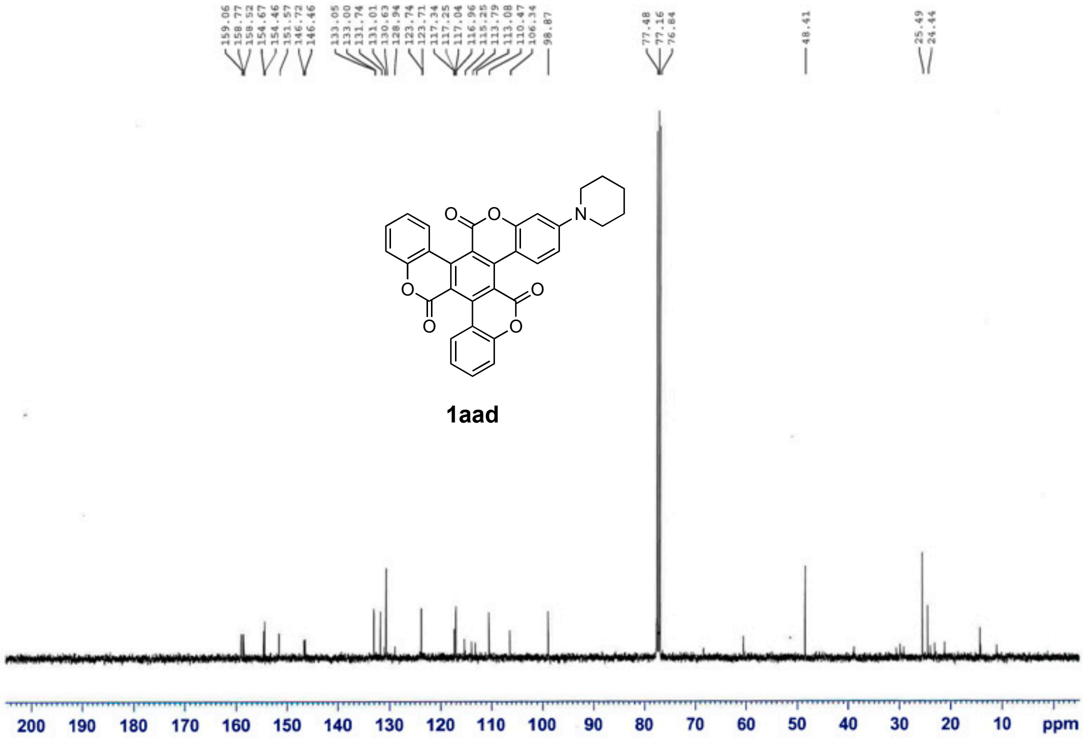

Figure S14. ^{13}C NMR spectrum of **1aad** (100 MHz, $CDCl_3$).

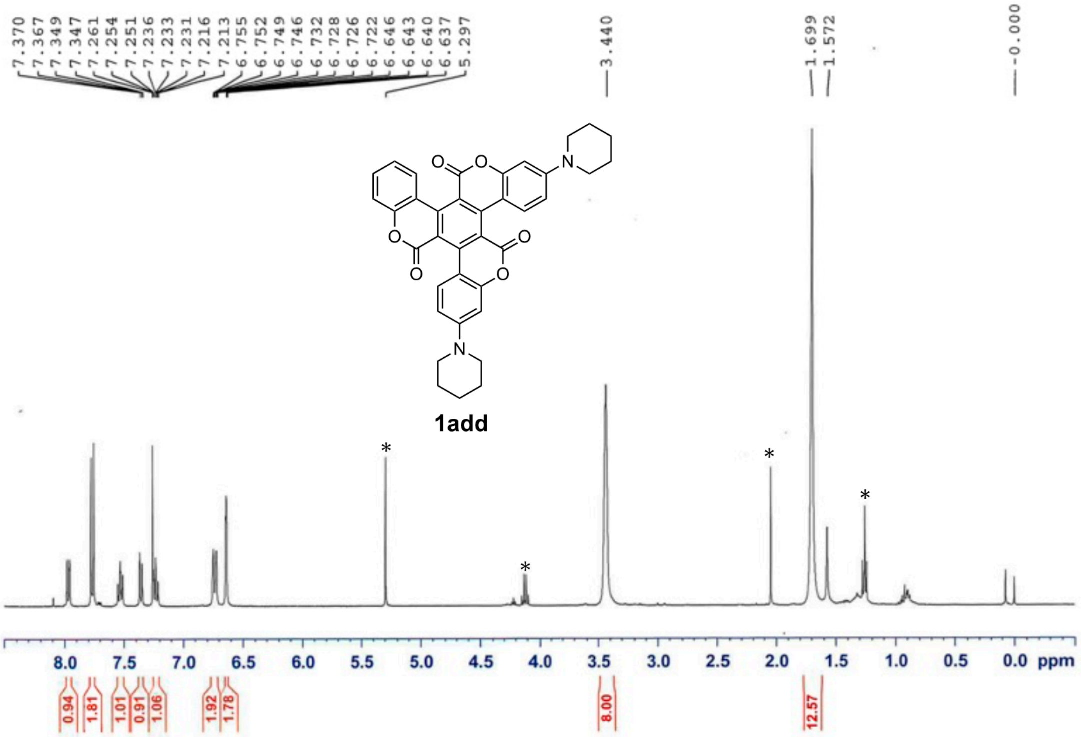

Figure S15. ^{1}H NMR spectrum of **1add** (400 MHz, $CDCl_3$, * = solvent peak).

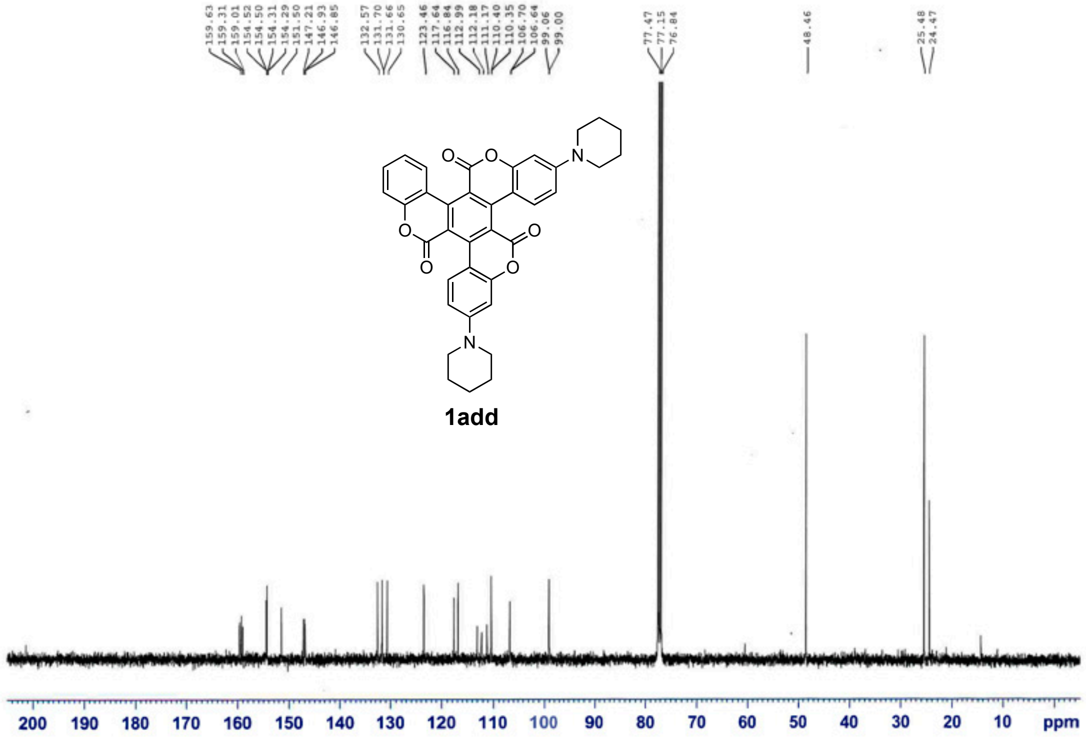

Figure S16. ^{13}C NMR spectrum of **1add** (100 MHz, $CDCl_3$).

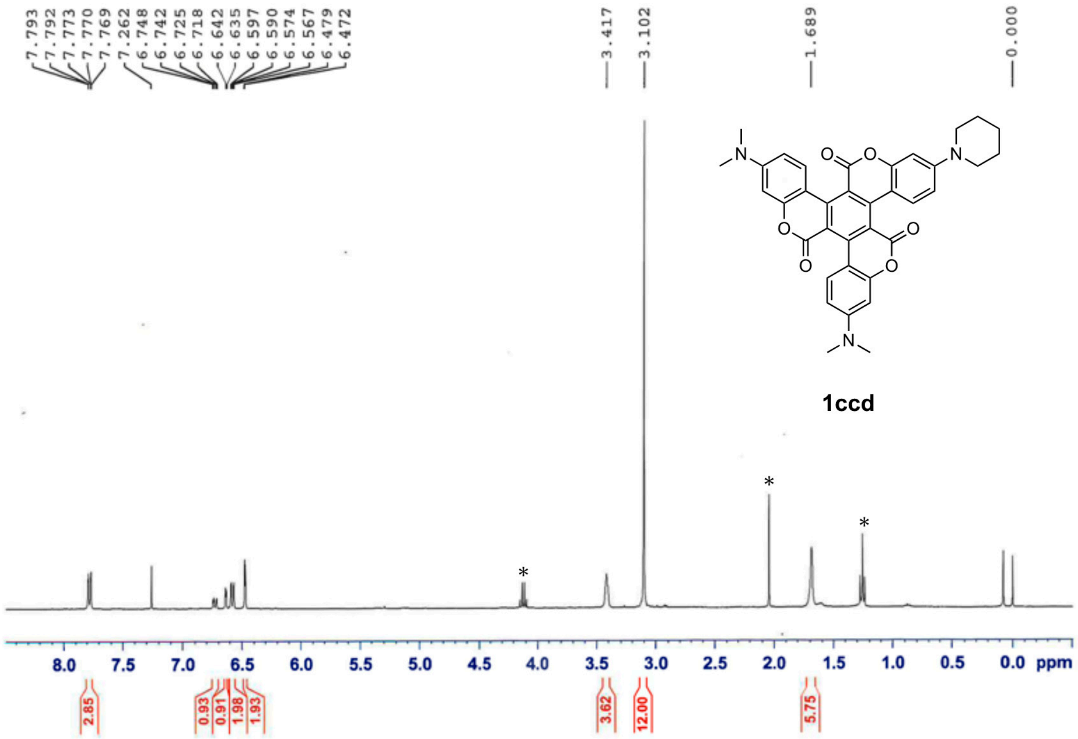

Figure S17. ^{1}H NMR spectrum of **1ccd** (400 MHz, $CDCl_3$, * = solvent peak).

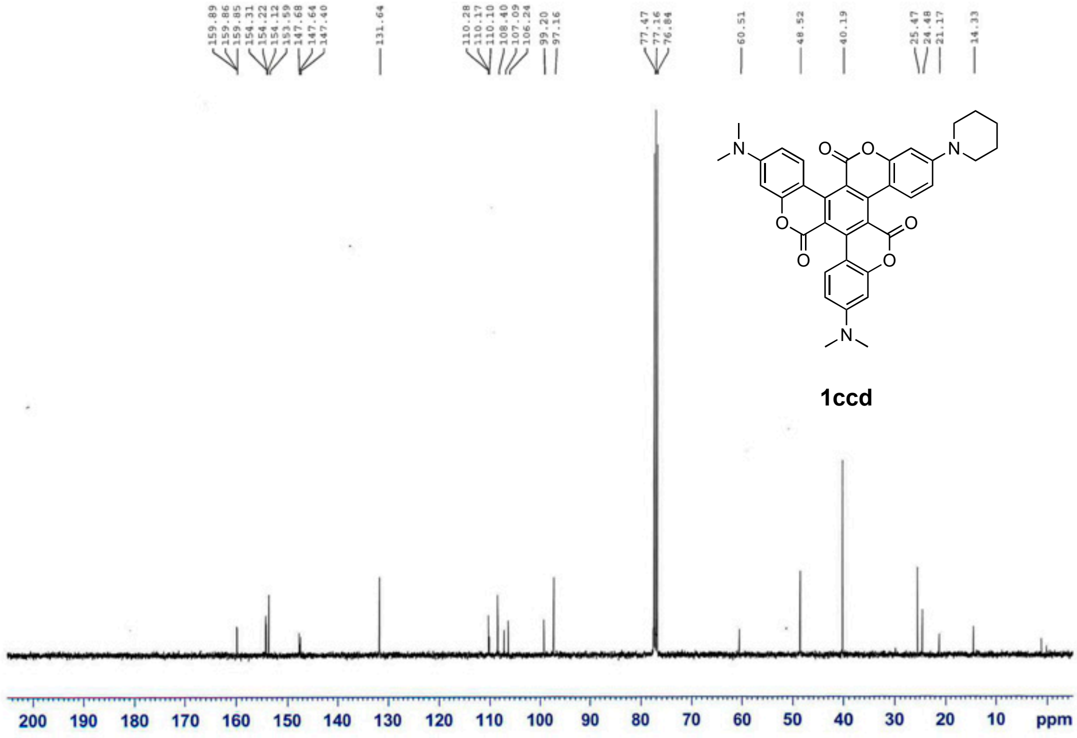

Figure S18. ^{13}C NMR spectrum of **1ccd** (100 MHz, $CDCl_3$).

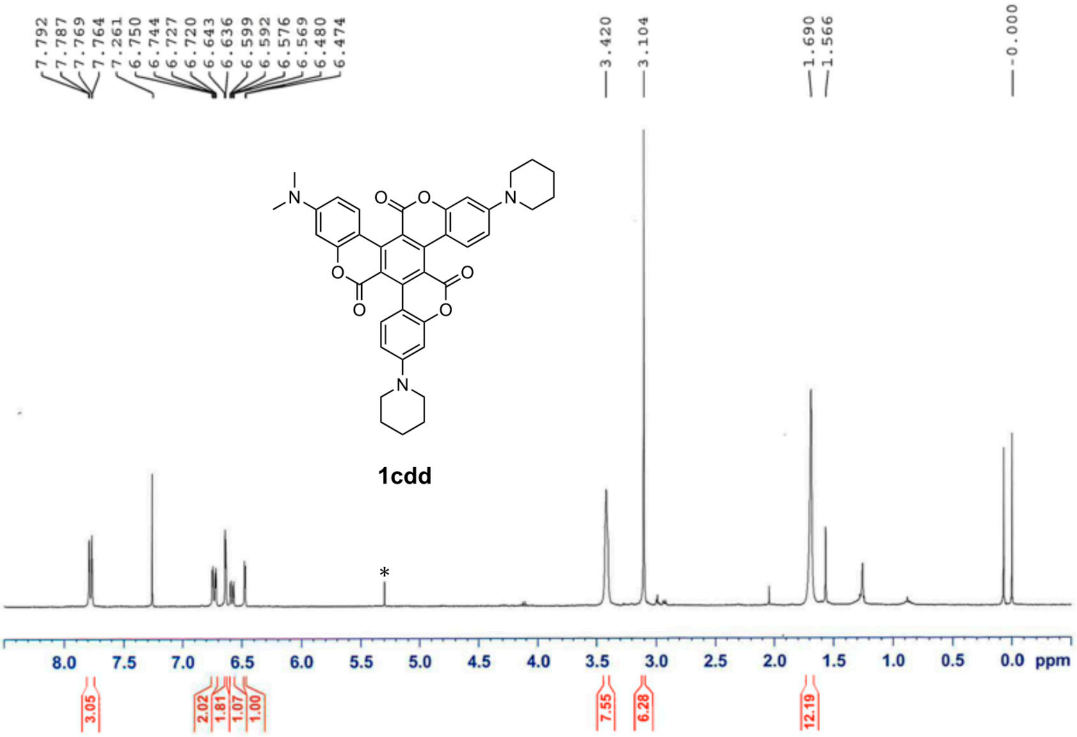

Figure S19. ^{1}H NMR spectrum of **1cdd** (400 MHz, $CDCl_3$, * = solvent peak).

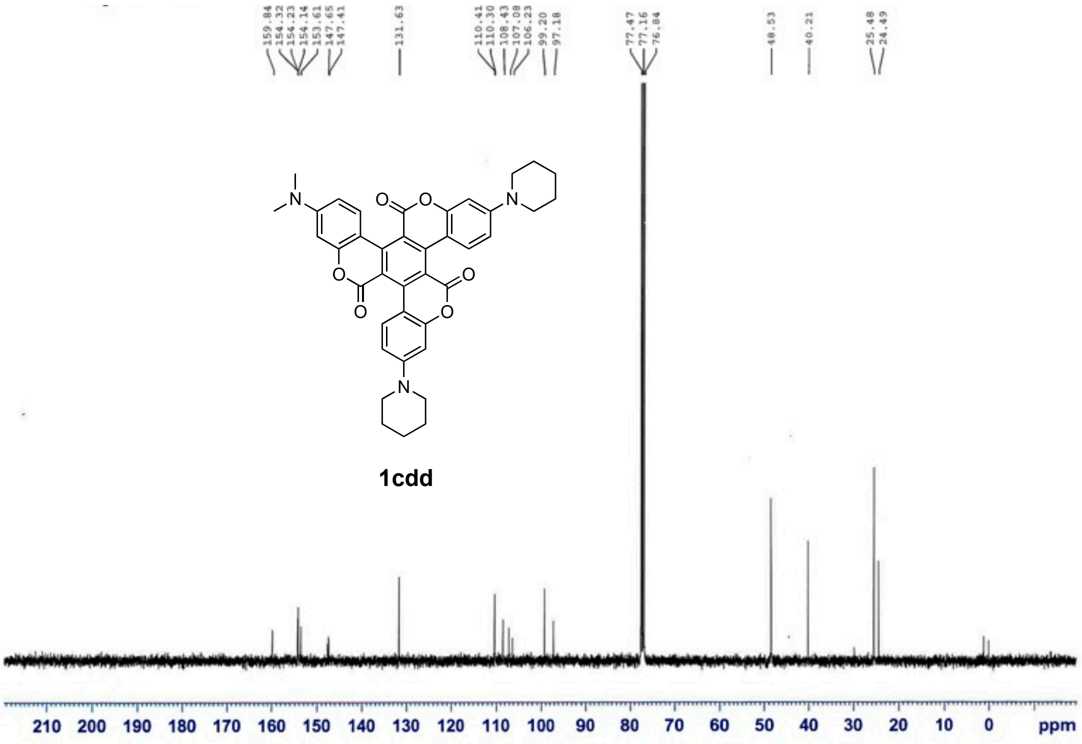

Figure S20. ^{13}C NMR spectrum of **1cdd** (100 MHz, $CDCl_3$).

2. Theretical Calculations

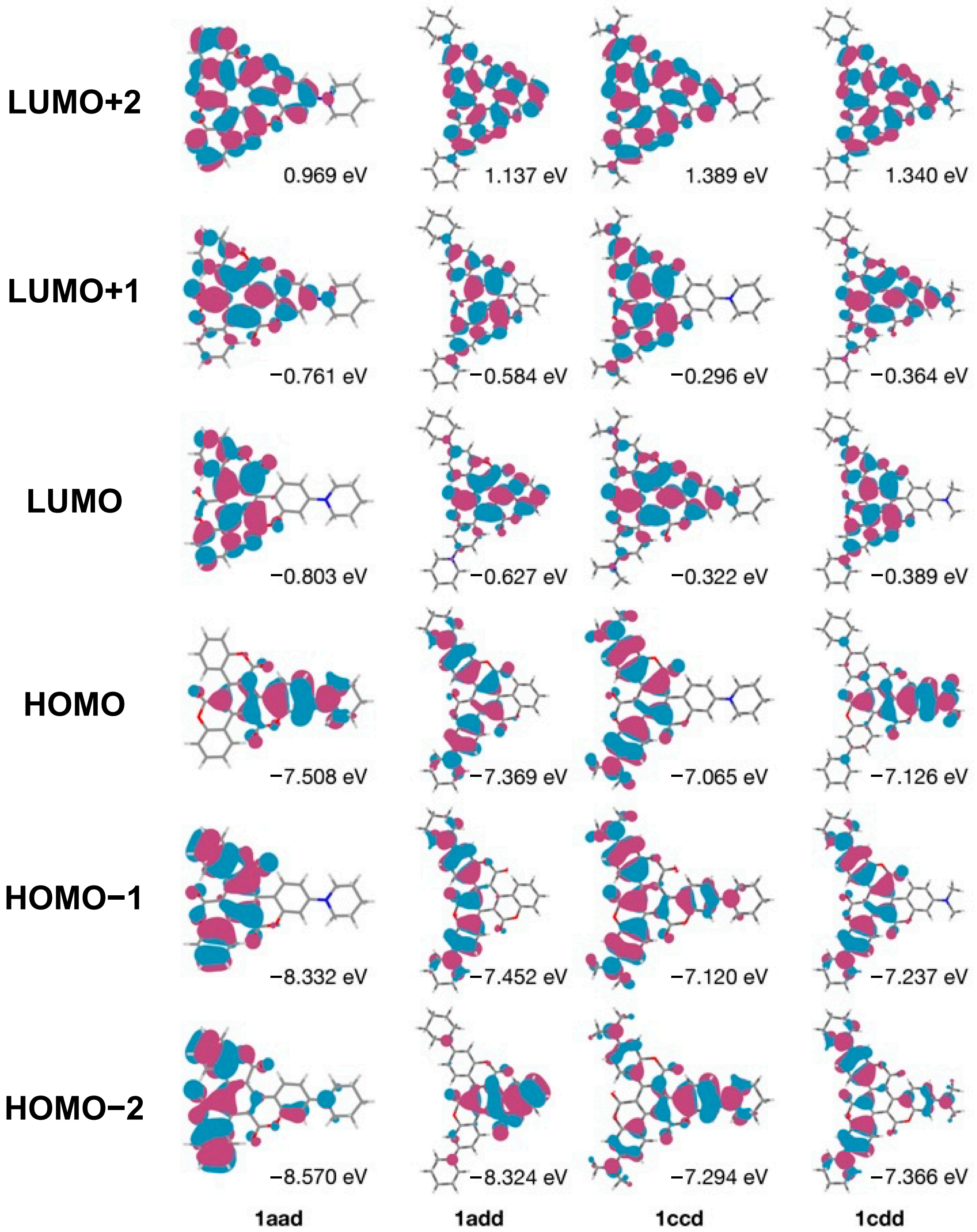

Figure S21. Molecular orbitals of **1aad**, **1add**, **1ccd**, and **1cdd** obtained by quantum chemical calculations at the ωB97-XD/6-31G(d) level.

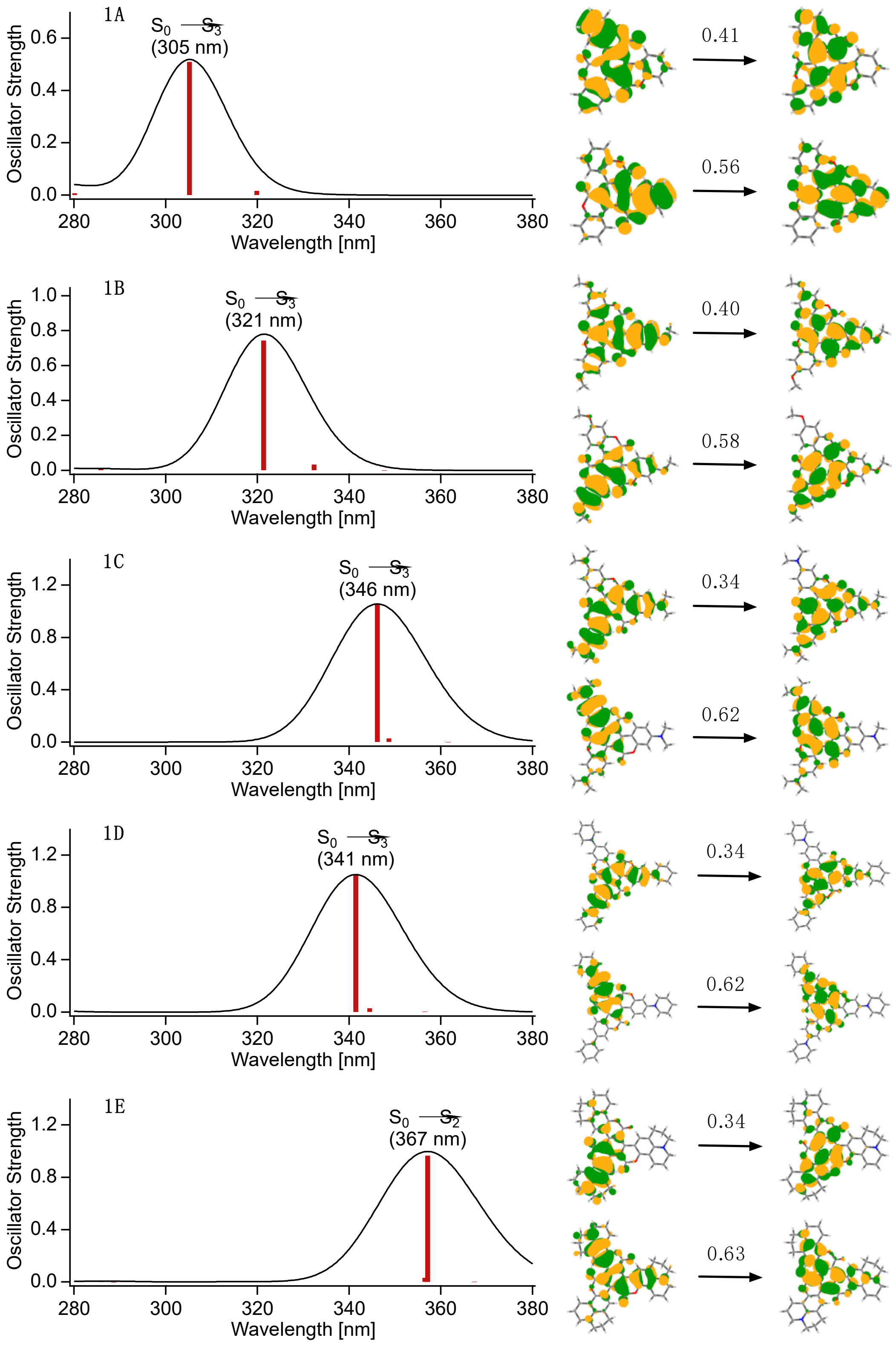

Figure S22. Absorption spectra simulated with quantum chemical calculations, and the main components of the natural transition orbitals (NTOs) for each electronic excitation. Absorption spectra were simulated by the Gaussian-shaped bands with a half width at half maximum of 1000 cm^{-1}.

3. Fluorescence Spectra

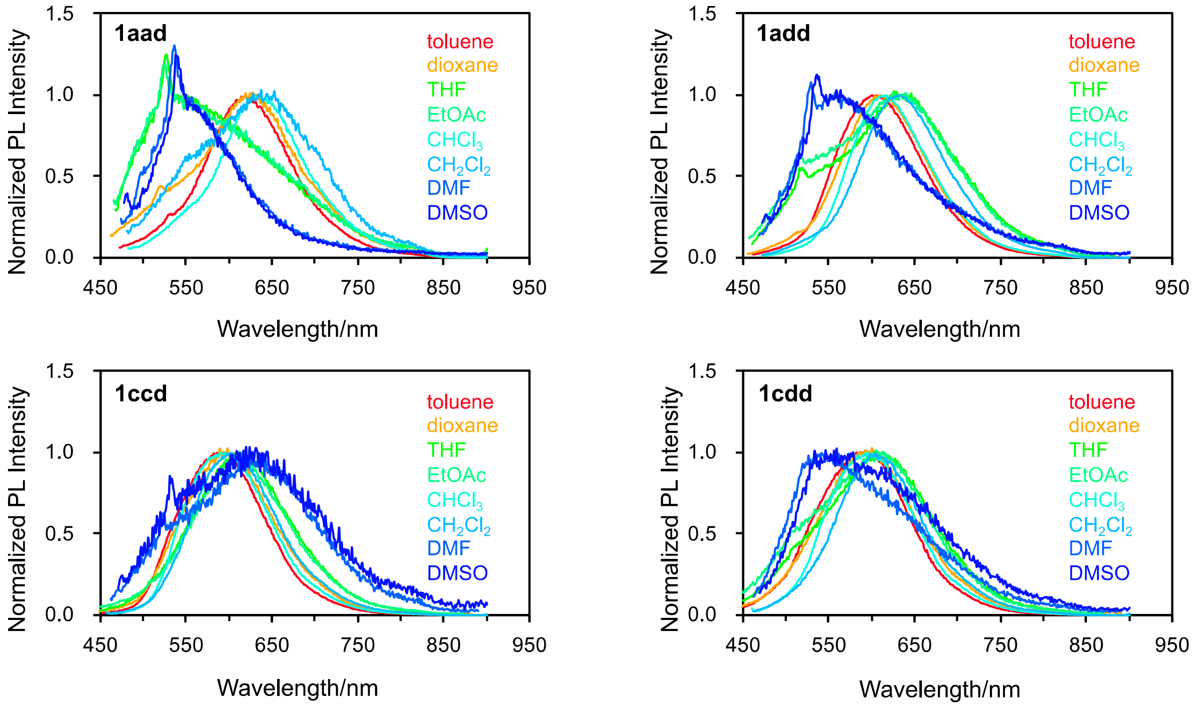

Figure S23. Emission spectra of **1aad**, **1add**, **1ccd**, and **1cdd** in several solvents.

Table S1. Photophysical properties of **1aad**, **1add**, **1ccd**, and **1cdd** in various solvents.

Compound	Solvent	λ_{abs}/nm	λ_{em}/nm	$\Delta S/cm^{-1}$	Φ_F
**1aad**	toluene	457	619	5727	0.006
	1,4-dioxane	452	630	6251	0.003
	THF	452	541	3640	0.001
	EtOAc	455	539	3425	0.001
	$CHCl_3$	473	634	5413	0.003
	CH_2Cl_2	472	636	5463	0.001
	DMF	464	548	3304	<0.001
	DMSO	466	545	3111	<0.001
**1add**	toluene	450	606	5720	0.007
	1,4-dioxane	445	609	6052	0.006
	THF	450	637	6523	0.001
	EtOAc	447	634	6598	0.001
	$CHCl_3$	464	616	5318	0.005
	CH_2Cl_2	463	634	5825	0.003
	DMF	458	560	3977	<0.001
	DMSO	463	542	3148	<0.001
**1ccd**	toluene	443	593	5710	0.003
	1,4-dioxane	440	593	5864	<0.001
	THF	443	617	6366	<0.001
	EtOAc	442	611	6317	<0.001
	$CHCl_3$	455	595	5171	0.002
	CH_2Cl_2	453	602	5464	0.001
	DMF	454	638	6352	0.001
	DMSO	460	627	5790	<0.001
**1cdd**	toluene	443	596	5794	0.003
	1,4-dioxane	440	601	6088	<0.001
	THF	443	612	6233	<0.001
	EtOAc	441	613	6363	<0.001
	$CHCl_3$	456	595	5123	0.001
	CH_2Cl_2	453	604	5519	<0.001
	DMF	453	542	3625	<0.001
	DMSO	459	534	3059	<0.001

4. Absorption spectra

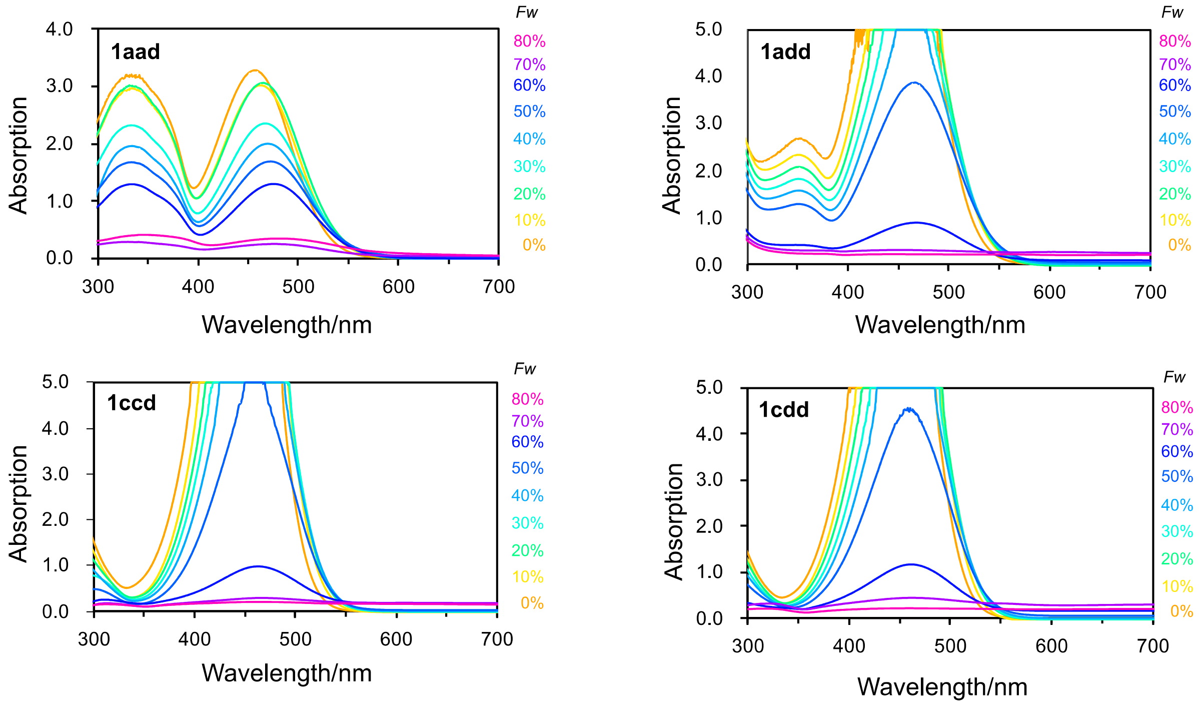

Figure S24. Absorption spectra of **1aad**, **1add**, **1ccd**, and **1cdd** in THF/H_2O mixtures.

5. DLS measurement

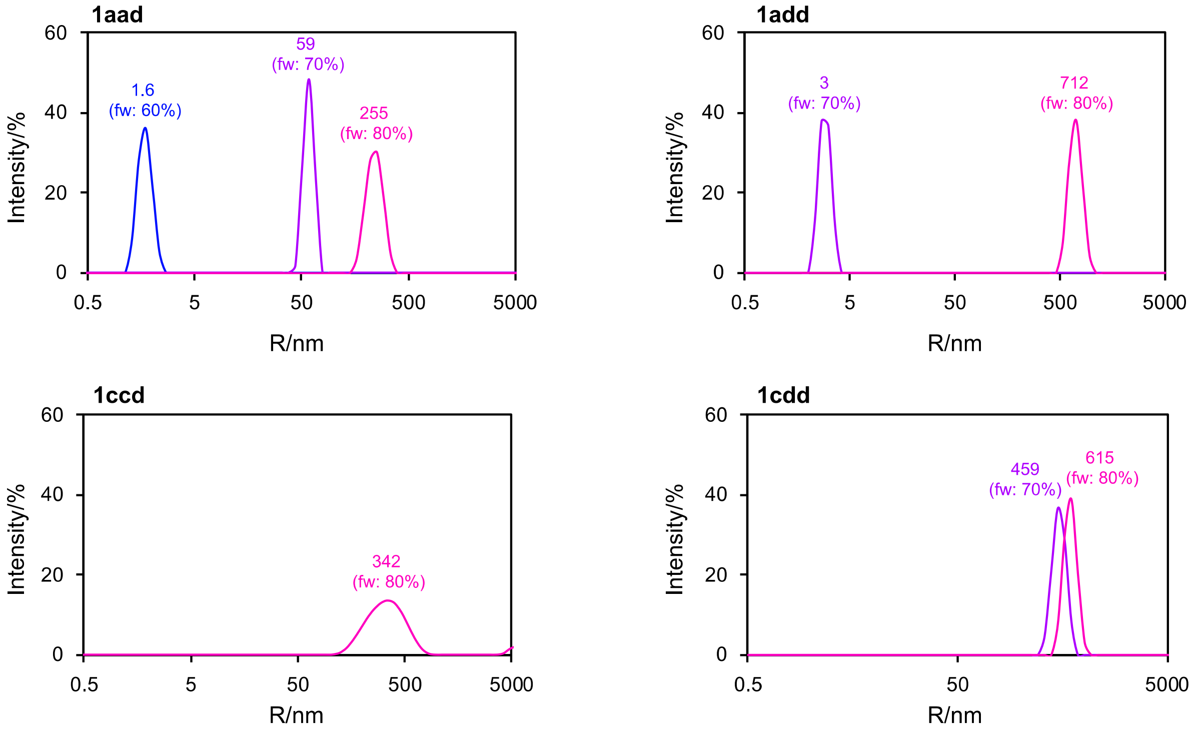

Figure S25. The size distributions based on THF/H_2O mixtures of DLS data for **1aad, 1add, 1ccd**, and **1cdd**.
